# Treatment of Steroid-Induced Osteonecrosis of the Distal Femoral Condyle Using Structural Bone Grafting

**DOI:** 10.2106/JBJS.OA.26.00089

**Published:** 2026-07-17

**Authors:** Dai Iwase, Yukie Metoki, Jun Aikawa, Manabu Mukai, Kensuke Fukushima, Kentaro Uchida, Gen Inoue, Masashi Takaso

**Affiliations:** 1 Department of Orthopedic Surgery, Kitasato University School of Medicine, Sagamihara City, Kanagawa, Japan

## Abstract

**Background:**

Steroid-induced osteonecrosis of the distal femoral condyle is an uncommon condition that predominantly affects young patients receiving high-dose corticosteroid therapy. Joint-preserving treatment options for precollapse lesions remain limited, particularly in cases with extensive subchondral involvement. The purpose of this study was to evaluate the clinical and radiological outcomes of structural bone grafting (SBG) using a bicortical iliac bone graft (autograft or allograft) for precollapse osteonecrosis of the distal femoral condyle.

**Methods:**

This retrospective case series included 11 knees in 8 patients with steroid-induced osteonecrosis of the distal femoral condyle who underwent SBG. All treated knees were classified as Association Research Circulation Osseous stage II. Clinical outcomes were assessed using the Japanese Orthopaedic Association (JOA) knee score, and radiological evaluation focused on graft incorporation and the presence of subchondral collapse or degenerative changes.

**Results:**

The mean follow-up duration was 150.5 months (48-288 months). No procedure-related complications were observed. Radiological graft incorporation was confirmed in all treated knees, and no progression to subchondral collapse or degenerative changes was identified during the observation period. The mean JOA knee score improved, and no deterioration was observed; 2 knees showed no change, 3 demonstrated modest improvement, and 6 showed greater improvement (15-40 points).

**Conclusions:**

SBG using a bicortical iliac bone graft provided favorable long-term clinical and radiological outcomes in this series of patients with precollapse steroid-induced osteonecrosis of the distal femoral condyle.

**Clinical Relevance:**

This technique may be useful for carefully selected patients with early-stage disease and extensive subchondral involvement.

**Level of Evidence:**

Level III, Therapeutic. See Instructions for Authors for a complete description of levels of evidence.

## Introduction

Steroid-induced osteonecrosis of the distal femoral condyle predominantly affects young patients receiving high-dose corticosteroid therapy for systemic diseases, creating a need for joint-preserving treatment strategies. In contrast to spontaneous osteonecrosis of the knee, which typically occurs in older individuals and often progresses rapidly to degenerative arthritis, secondary osteonecrosis in younger patients raises important concerns regarding long-term joint preservation^1-4^.

For precollapse lesions, core decompression has been widely accepted as a joint-preserving option, with generally favorable outcomes for early-stage disease^5^. The aim of core decompression was to reduce intraosseous pressure and promote revascularization; however, disease stage alone does not necessarily reflect lesion characteristics relevant to structural durability. In osteonecrosis of the distal femoral condyle, lesion size and the extent of femoral condylar involvement have been associated with subsequent structural deterioration and progression to osteoarthritic changes, indicating that factors beyond stage classification should be considered when selecting treatment strategies^6,7^.

Bone grafting techniques combined with curettage have therefore been introduced. Autologous cancellous iliac bone grafting improves clinical symptoms; however, its ability to provide durable structural support may be limited in certain lesion configurations^8,9^. In osteonecrosis of the femoral head, structural bone grafting (SBG) techniques were developed to restore subchondral load-bearing architecture in precollapse disease^10-12^. Based on this biomechanical concept, SBG may also be applicable to selected cases of precollapse steroid-induced osteonecrosis of the distal femoral condyle. The aim of this study was to evaluate the clinical and radiological outcomes of SBG using a bicortical iliac bone graft for steroid-induced osteonecrosis of the distal femoral condyle.

## Materials and Methods

### Study Population

**Fig. 1:**
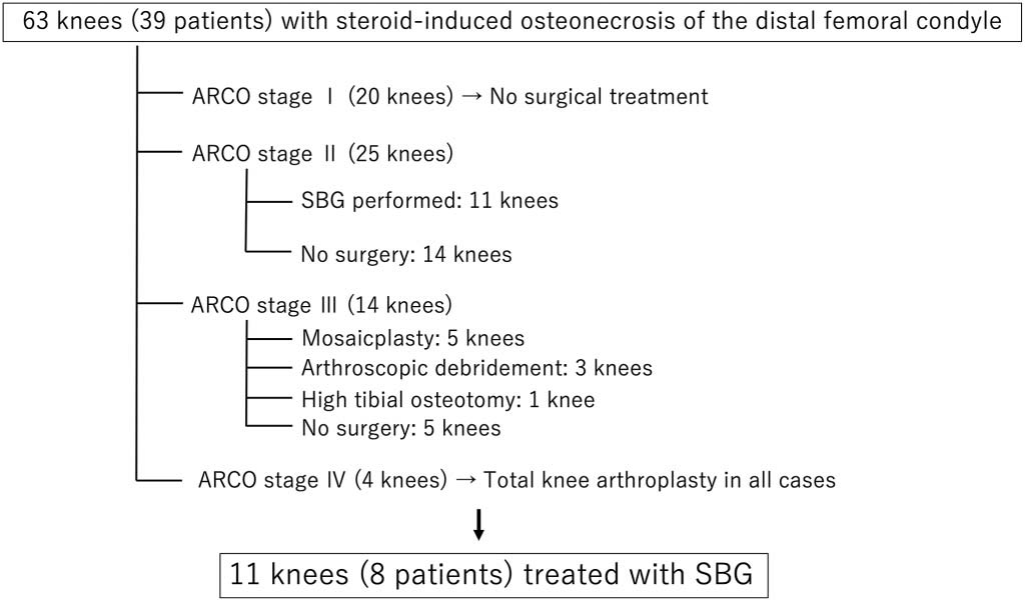
Flow diagram of patient selection. During the study period, 63 knees in 39 patients with steroid-induced osteonecrosis of the distal femoral condyle were identified. Treatment strategies were determined based on clinical presentation and disease severity. All ARCO stage I knees were managed nonoperatively. Among ARCO stage II knees, SBGs were performed in 11 knees, while the remaining knees were treated conservatively. ARCO stage III knees were managed with mosaicplasty, arthroscopic debridement, high tibial osteotomy, or nonoperative treatment. All ARCO stage IV knees were treated with total knee arthroplasty. Ultimately, 11 knees in 8 patients treated with SBG were included in the final analysis. ARCO = Association Research Circulation Osseous, and SBG = structural bone grafting.

This retrospective study included 11 knees in 8 patients who underwent SBG for steroid-induced osteonecrosis of the distal femoral condyle at a single institution between January 1994 and January 2022, with a minimum follow-up of 4 years. During the study period, 63 knees in 39 patients with steroid-induced osteonecrosis of the distal femoral condyle were identified. SBGs were performed in selected symptomatic cases, and the patient selection process is summarized in Fig. 1. All patients were female, with a mean age of 28.5 years (range, 14-54 years) at the time of surgery. Race and ethnicity data were not collected as part of this retrospective study conducted at a single institution in Japan.

### Radiological Evaluation and Staging

Preoperative evaluation was performed using plain radiographs and magnetic resonance imaging (MRI). Disease staging was determined according to the Association Research Circulation Osseous (ARCO) classification system^13^. Because the ARCO classification was originally developed for osteonecrosis of the femoral head and has not been validated as a prognostic staging system for osteonecrosis of the knee, it was used in this study only as a descriptive framework to indicate precollapse disease. ARCO staging was assigned retrospectively and was not used to determine surgical indication. Surgical indication was determined clinically based on symptoms, lesion location on MRI, and radiographic confirmation of precollapse disease. Lesion size and depth from the subchondral bone were also assessed retrospectively to describe lesion characteristics. All treated knees were classified as ARCO stage II, defined by radiographic evidence of sclerosis and/or cystic changes without subchondral collapse.

In addition to ARCO staging, lesion size and location were evaluated on preoperative MRI. The ratio of lesion width to condylar width (L/C) was measured on coronal images at the level of maximal lesion extent, and sagittal lesion length (Sag L) and depth from the subchondral bone (Sag D) were measured on sagittal images. Lesions were considered extensive when they involved a substantial proportion of the condylar width (generally >40%-50%) and extended along the weight-bearing region. Proximity to the subchondral plate was also considered, and all lesions were located immediately beneath or in close proximity to the subchondral plate. These measurements were performed retrospectively.

Postoperative radiographic evaluation was performed using serial plain radiographs and/or computed tomography (CT) at each follow-up visit. Graft incorporation was defined as the presence of trabecular continuity between the graft and host bone without radiolucent lines. Subchondral collapse was defined as any new depression or flattening of the articular surface on follow-up imaging compared with preoperative findings. Degenerative changes were defined as the progression of joint space narrowing, osteophyte formation, or subchondral sclerosis. All imaging assessments were performed by 2 observers, with disagreements resolved by consensus.

### Surgical Procedures and Postoperative Management

On MRI, osteonecrotic lesions were frequently observed on both the medial and lateral femoral condyles. When multiple osteonecrotic lesions were present, the symptomatic site was determined clinically based on pain location and corresponding lesion location on MRI. Arthroscopic evaluation was performed first to assess the articular cartilage, and the absence of cartilage delamination or fissuring was confirmed.

Subsequently, a Kirschner wire was placed on the skin surface, and fluoroscopic imaging was used to confirm that the necrotic lesion could be accessed via an extra-articular approach before making the skin incision. A cortical window was then created, and curettage of the necrotic bone was performed through this window primarily under fluoroscopic guidance. When the lesion was not clearly identifiable on fluoroscopy alone, preoperative MRI findings were used to assist in localization. This was followed by placement of a bicortical iliac bone graft beneath the subchondral plate to provide structural support. Additional technical details are provided in the Appendix (Supplementary Fig. 1).

Postoperatively, patients were initially managed with non–weight bearing, followed by gradual progression to full weight bearing. Rehabilitation protocols evolved over time, with earlier progression of weight bearing adopted once the procedure was confirmed safe (Table I).

**TABLE I:** Baseline Characteristics of Patients and Treated Knees

Patient No.	Side	Age (Years)	BMI (kg/m^2^)	Gender	Diagnosis	Treated site	ARCO	Graft type	L/C (%)	Sag L (mm)	Sag D (mm)	Fixation	PO Rehabilitation
1	R	14	14.8	Female	SLE	L	Ⅱ	Autograft	44.1	36.5	9.9	Yes	NWB 6 weeks → FWB
2	R	33	18.5	Female	SLE	L	Ⅱ	Autograft	56.0	28	13	Yes	NWB 6 weeks → FWB
	L	33	18.5	Female	SLE	M	Ⅱ	Autograft	54.3	30.6	12.5	Yes	NWB 6 weeks → FWB
3	R	19	21.8	Female	MCTD	M	Ⅱ	Autograft	51.9	35.8	11.2	Yes	NWB 6 weeks → FWB
	L	21	21.8	Female	MCTD	M	Ⅱ	Autograft	47.2	24.3	10.7	Yes	NWB 6 weeks → FWB
4	L	24	24.1	Female	SLE	M	Ⅱ	Allograft	49.1	28.1	9.2	Yes	NWB 3 weeks → FWB
5	L	23	19.4	Female	SLE	M	Ⅱ	Allograft	57.0	20.3	11.2	Yes	NWB 3 weeks → FWB
						L	Ⅱ	Allograft	60.3	28.8	13.7	Yes	
	R	25	19.4	Female	SLE	M	Ⅱ	Allograft	52.3	23.4	12.7	Yes	NWB 3 weeks → FWB
6	R	37	22.0	Female	MCTD	L	Ⅱ	Allograft	56.3	42.6	32.3	No	NWB 3 weeks → FWB
7	R	35	24.7	Female	Drug eruption*	M	Ⅱ	Allograft	41.6	23.8	10.7	No	NWB 1 week → FWB
8	R	54	30.8	Female	SSc	M	Ⅱ	Allograft	40.4	26.3	10.1	No	PO day 2 → FWB
						L	Ⅱ	Allograft	53.3	20.1	9.1	No	

ARCO = Association Research Circulation Osseous Classification, BMI = body mass index, FWB = full weight bearing, L = lateral, L/C = ratio of lesion width to condylar width measured on coronal MRI at the level of the maximal lesion extent, M = medial, MCTD = mixed connective tissue disease, ML = medial and lateral, MRI = magnetic resonance imaging, NWB = non–weight bearing, PO = postoperative, PWB = partial weight bearing, Sag D = maximal sagittal depth of the osteonecrotic lesion measured from the subchondral bone on MRI, Sag L = maximal sagittal depth of the osteonecrotic lesion measured on MRI, SLE = systemic lupus erythematosus, and SSc = systemic sclerosis

* Lamotrigine-induced drug eruption requiring steroid pulse therapy.

Arrows (→) indicate progression of weight-bearing status.

Among the cases included in this study, all asymptomatic knees that were not treated surgically were classified as ARCO stage Ⅰ or Ⅱ. These knees were managed nonoperatively because they remained asymptomatic. However, detailed longitudinal radiological evaluation of untreated asymptomatic lesions was not performed systematically; therefore, their natural history could not be determined.

### Outcome Assessment

Radiological outcomes included graft incorporation and the presence or absence of subchondral collapse or degenerative changes within 4 years postoperatively.

Clinical outcomes were evaluated using the Japanese Orthopaedic Association (JOA) knee score^14^. The JOA score was assessed preoperatively and at final follow-up.

### Statistical Analysis

Changes in JOA knee scores between preoperative and final follow-up evaluations were analyzed using the Wilcoxon signed rank test. Data are presented as mean ± SD. A p value of < 0.05 was considered statistically significant. Because some patients contributed bilateral knees, the unit of analysis was the knee, and the observations were not fully independent. Given the small sample size, no adjustment for within-patient correlation was performed.

## Results

The mean duration of follow-up was 150.5 months (48-288 months). No procedure-related complications were observed. Radiologically, graft incorporation was confirmed in all treated knees. Clinically, the mean total JOA knee score showed significant improvement from 68.2 ± 8.6 points preoperatively to 82.3 ± 14.2 points at the final follow-up (p = 0.0039). The mean increase in JOA score was 14.1 points (95% confidence interval, 5.4-22.8), with no deterioration observed; 2 knees showed no change, 3 demonstrated modest improvement, and 6 showed greater improvement (15-40 points). Individual clinical outcomes, radiological findings, including graft union, and follow-up duration for each treated knee are summarized in Table II.

**TABLE II:** Clinical and Radiographic Outcomes of the Treated Knees

				Pre JOA					Post JOA				
Patient No.	Side	Follow-up (Months)	Bone Union	Total	Gait	Step	ROM	Swelling	Total	Gait	Step	ROM	Swelling
1	R	288	+	70	15	15	30	10	75	20	15	30	10
2	R	276	+	60	15	10	35	0	60	15	10	35	0
	L	276	+	60	15	10	35	0	60	15	10	35	0
3	R	232	+	85	25	15	35	10	100	30	25	35	10
	L	208	+	70	15	10	35	10	100	30	25	35	10
4	L	142	+	80	20	20	30	10	85	25	20	30	10
5	L	104	+	60	15	10	30	5	100	30	25	35	10
	R	70	+	65	15	10	30	10	90	25	20	35	10
6	R	98	+	75	15	15	35	10	80	20	15	35	10
7	R	51	+	65	15	10	30	10	80	25	15	30	10
8	R	48	+	60	15	5	30	10	75	25	10	30	10

JOA = Japanese Orthopaedic Association, ROM = range of motion, R = right, and L = left.

Bone union was evaluated radiologically using plain radiographs and/or computed tomography.

### Case Presentation (Patient No. 7, Right Knee)

**Fig. 2:**
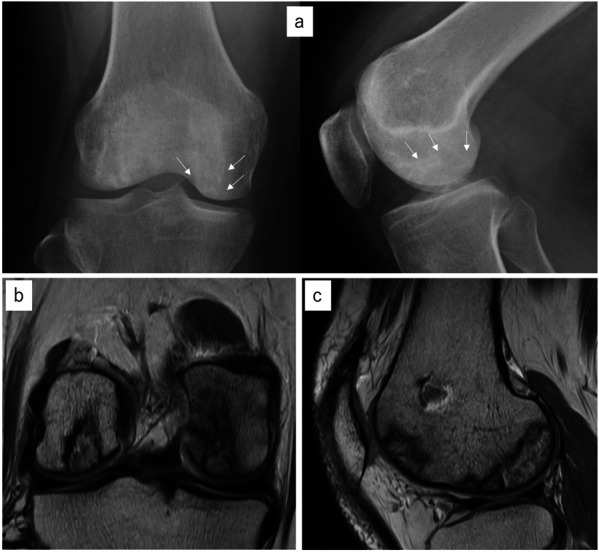
Preoperative imaging findings of the case (Patient No. 7, right knee). **Fig. 2-A** Anteroposterior and lateral plain radiographs demonstrating focal sclerotic changes in the medial femoral condyle (white arrows). **Fig. 2-B** Coronal proton density-weighted MRI showing osteonecrotic lesions involving the weight-bearing regions of both femoral condyles. **Fig. 2-C** Sagittal proton density-weighted MRI of the medial femoral condyle demonstrating extensive osteonecrosis in the posterior aspect of the condyle. MRI = magnetic resonance imaging.

A representative case is presented. A 35-year-old woman had a history of hospitalization for a severe lamotrigine-induced drug eruption, for which she received steroid pulse therapy at another institution; detailed information regarding the pulse regimen was unavailable, and no subsequent oral prednisolone therapy had been administered. Five years after the pulse therapy, she presented to our institution with right knee pain. Her pain during walking and stair climbing was localized medially. The preoperative JOA knee score was 65 points. Plain radiographs demonstrated sclerotic changes with cystic lesions in the medial femoral condyle. MRI revealed an extensive osteonecrotic lesion in the posterior portion of the medial femoral condyle; a lateral condylar lesion was also present but was considered asymptomatic. The articular cartilage surface was intact (Fig. 2).

**Fig. 3:**
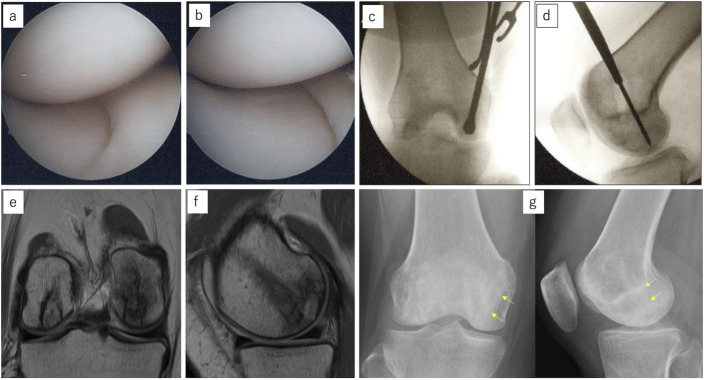
Intraoperative and postoperative findings of the case (Patient No. 7, right knee). **Fig. 3-A** Arthroscopic view at 90° of knee flexion obtained before curettage, demonstrating an intact articular cartilage surface without fissuring or delamination. **Fig. 3-B** Arthroscopic view in deep knee flexion obtained before curettage, confirming preservation of the cartilage surface in the posterior aspect of the medial femoral condyle. **Figs. 3-C and 3-D** In this case, drilling toward the necrotic lesion was performed using a cannulated drill under fluoroscopic guidance to facilitate access to the subchondral region, followed by use of an expandable reamer (9-mm diameter) to further evacuate necrotic bone, and subsequent curettage. Arthroscopic evaluation was performed after debridement to confirm that no iatrogenic damage to the articular surface had occurred. **Figs. 3-E and 3-F** Postoperative proton density-weighted magnetic resonance imaging obtained 6 months after surgery showing preservation of the articular surface without evidence of subchondral collapse. **Fig. 3-G** Plain radiograph obtained at the final follow-up (4 years postoperatively) demonstrating graft incorporation (yellow arrows) without progression of subchondral collapse. On this final follow-up radiograph, the untreated lateral femoral condylar lesion, which was considered asymptomatic preoperatively, showed no obvious radiographic progression or subchondral collapse during the 4-year follow-up period.

SBG was performed for the symptomatic medial femoral condyle. Arthroscopic examination confirmed the absence of cartilage fissuring. Under fluoroscopic guidance, drilling was performed using a cannulated drill up to the subchondral region, followed by thorough curettage of the surrounding necrotic bone, and SBG using a bicortical iliac bone allograft was completed. At the final follow-up 4 years and 3 months after surgery, the JOA knee score had improved to 80 points. Radiographs and MRI demonstrated graft incorporation, with preservation of the articular surface and no evidence of subchondral collapse in the treated medial femoral condyle; the untreated lateral condylar lesion also showed no apparent collapse or obvious radiological progression and remained at the precollapse stage (Fig. 3).

## Discussion

This study showed that SBG using a bicortical iliac bone graft resulted in consistent graft incorporation and favorable long-term outcomes in precollapse steroid-induced osteonecrosis of the distal femoral condyle. No procedure-related complications were observed, and radiographic progression to subchondral collapse or degenerative changes was not identified at the final follow-up.

Joint-preserving procedures are generally recommended for precollapse osteonecrosis, and core decompression yields favorable outcomes in early-stage disease^5^. These findings support its use in selected patients. However, previous studies have also indicated that lesion extent plays an important role in prognosis. Long-term observational studies and reviews of femoral condylar osteonecrosis have shown that larger lesions are associated with subsequent structural deterioration and progression to osteoarthritis, suggesting that disease stage alone may not fully capture mechanical risk^6,7^.

Curettage combined with cancellous bone grafting improves clinical symptoms; however, prior studies of steroid-induced osteonecrosis of the distal femoral condyle have indicated that cancellous bone grafting does not always prevent subsequent subchondral collapse despite symptomatic improvement^8,9^. These findings suggest that cancellous grafting alone may be insufficient to provide durable subchondral support in certain lesion configurations.

These findings suggest that disease stage alone may not guide treatment selection in osteonecrosis of the distal femoral condyle. All treated knees were retrospectively classified as ARCO stage Ⅱ; however, ARCO staging itself was not the basis for surgical indication. Surgical indication was determined clinically based on symptoms, lesion location on MRI, and radiographic confirmation of precollapse disease. Retrospective MRI assessment showed that many lesions were extensive, involved approximately 40% to 50% of the condylar width, and were located close to the subchondral bone; however, these measurements were not used prospectively for surgical decision making. In such cases, decompression alone may not adequately address subchondral mechanical vulnerability. Although no definitive conclusions can be drawn without a control group, these findings suggest that SBG may be considered for carefully selected extensive subchondral lesions.

In contrast to cancellous grafting, the present technique used a bicortical iliac bone graft shaped as a structural strut and impacted close to the subchondral plate. This approach was designed to restore load-bearing architecture beneath the articular surface while preserving native cartilage, which was confirmed arthroscopically in all cases. Although the femoral condyle differs anatomically from the femoral head, the principle of augmenting debridement with structural support has long been explored in femoral head osteonecrosis for preventing collapse in precollapse disease^10-12^.

The present technique is not intended to replace core decompression but to extend joint-preserving surgery in selected cases. While decompression alone may be sufficient for small or centrally located lesions, SBG may offer an additional mechanical advantage when necrotic involvement is extensive or closely associated with the weight-bearing subchondral plate. This concept may help refine surgical decision making beyond disease stage alone.

More invasive reconstructive procedures, such as osteochondral autograft transfer or fresh osteochondral allograft transplantation, have demonstrated favorable outcomes but are generally reserved as salvage options when the articular surface is compromised^15-17^. By contrast, the present approach aims to preserve the native cartilage while reinforcing the underlying subchondral structure.

This study has several limitations. First, race and ethnicity data were not collected, limiting the generalizability of the findings to more diverse populations. In addition, all patients were female. This was not an intentional selection criterion, but rather reflected the characteristics of the cases encountered during the study period. Therefore, generalizability to male patients remains uncertain. Second, the unit of analysis was the knee rather than the patient, and some patients contributed bilateral knees, which may have violated the assumption of independent observations. Third, the treatment protocol was not fully uniform across cases. Although the procedure was described as SBG, variations existed in graft source (autograft or allograft), use of screw fixation, and postoperative rehabilitation protocols, which also evolved over time. These factors may influence both clinical and radiographic outcomes and complicate interpretation. Descriptive stratification by graft source and fixation showed no obvious differences, but the small sample size precludes meaningful subgroup comparisons. Rehabilitation protocols also evolved, although no clear treatment-era effect was identified. Fourth, osteonecrotic lesions were sometimes multifocal, and symptoms may not have originated solely from the treated lesion. Although the symptomatic site was determined clinically based on pain location and corresponding lesion location on MRI, the contribution of each lesion, including possible osteonecrosis at other sites such as the hip, could not be determined definitively. Untreated asymptomatic knee lesions showed no obvious progression or collapse but were not evaluated systematically; therefore, their natural history remains unclear. Finally, the retrospective design, very small sample size, treatment heterogeneity, inclusion of bilateral knees, and absence of a control group limit definitive conclusions; therefore, these findings should be interpreted as preliminary evidence.

Despite these limitations, long-term follow-up and consistent graft incorporation suggest that SBG using a bicortical iliac bone graft may be a useful joint-preserving option for carefully selected patients with precollapse steroid-induced osteonecrosis of the distal femoral condyle.

## Author’s Contributions

D. Iwase conceptualized and designed the study, performed data acquisition and analysis, and drafted the manuscript. Y. Metoki and J. Aikawa contributed to data acquisition and interpretation of clinical and radiological findings. M. Mukai, K. Fukushima, and K. Uchida assisted with data collection and critically reviewed the manuscript. G. Inoue and M. Takaso supervised the study and contributed to the interpretation of the results. All authors reviewed and approved the final manuscript.

## Ethical Approval

This study was approved by the institutional review board of the Kitasato University Hospital (approval number B22-216) and performed in accordance with the ethical standards laid down in the 1964 Declaration of Helsinki and its later amendments. The board waived the requirement for informed consent because of the retrospective study design.

## Consent for Publication

Not applicable.

## Availability of Data and Materials

The data sets supporting the conclusions of this article are included within the article. The corresponding author can provide raw data on request.

## Competing Interests

All authors declare that they have no competing interests.

## Funding

None.

## Appendix

Supporting material provided by the authors is posted with the online version of this article as a data supplement at jbjs.org (http://links.lww.com/JBJSOA/B286). This content was not copyedited or verified by JBJS.

## References

[1] Mont MA Hungerford DS. Non-traumatic avascular necrosis of the femoral head. J Bone Jt Surg Am. 1995;77(3):459-74. 10.2106/00004623-199503000-00018 7890797

[2] Jones JP Jr. Intravascular coagulation and osteonecrosis. Clin Orthop Relat Res. 1992;277:41-53. 1532547

[3] Ahlbäck S Bauer GC Bohne WH. Spontaneous osteonecrosis of the knee. Arthritis Rheum. 1968;11(6):705-33. 5700639 10.1002/art.1780110602

[4] Yamamoto T Bullough PG. Spontaneous osteonecrosis of the knee: the result of subchondral insufficiency fracture. J Bone Jt Surg Am. 2000;82(6):858-66. 10.2106/00004623-200006000-00013 10859106

[5] Jacobs MA Hungerford DS Krackow KA Lennox DW. Osteonecrosis of the knee treated by core decompression. J Bone Jt Surg Br. 1989;71:524-9.

[6] Juréus J Lindstrand A Geijer M Robertsson O Tägil M. The natural course of spontaneous osteonecrosis of the knee (SPONK): a 1- to 27-year follow-up of 40 patients. Acta Orthop. 2013;84(4):410-4. 23799344 10.3109/17453674.2013.810521 PMC3768043

[7] Karim AR Cherian JJ Jauregui JJ Pierce T Mont MA. Osteonecrosis of the knee: review. Ann Transl Med. 2015;3(1):6. 25705638 10.3978/j.issn.2305-5839.2014.11.13 PMC4293480

[8] Murakami H Ochi M Uchio Y Adachi N Iwasa J. Curettage and autogenous bone grafting for steroid-related osteonecrosis of the femoral condyle. Clin Orthop Relat Res. 2004(421):239-46.

[9] Mont MA Marker DR Zywiel MG Carrino JA. Osteonecrosis of the knee and related conditions. Am Acad Orthop Surg. 2011;19(8):482-94. 10.5435/00124635-201108000-00004 21807916

[10] Phemister DB. Treatment of the necrotic head of the femur in adults. J Bone Jt Surg. 1949;31(1):55-66. 18106749

[11] Marcus ND Enneking WF Massam RA. The silent hip in idiopathic aseptic necrosis: treatment by bone-grafting. J Bone Jt Surg. 1973;55(7):1351-66. 4758708

[12] Mont MA Jones LC Seyler TM Marulanda GA Saleh KJ Delanois RE. New treatment approaches for osteonecrosis of the femoral head. Instr Course Lect. 2007;56:197-212. 17472307

[13] ARCO Committee on Terminology and Classification. The 2019 revised ARCO classification for osteonecrosis of the femoral head. J Arthroplasty. 2019;34:111-8.

[14] Japanese Orthopaedic Association. Criteria for evaluation of knee disorders [in Japanese]. J Jpn Orthop Assoc. 1988;62:901-9.

[15] Hangody L Füles P. Autologous osteochondral mosaicplasty for the treatment of full-thickness defects of weight-bearing joints: ten years of experimental and clinical experience. J Bone Jt Surg Am. 2003;85(suppl 2):25-32. 10.2106/00004623-200300002-00004 12721342

[16] Gross AE Kim W Las Heras F Backstein D Safir O Pritzker KP. Fresh osteochondral allografts for posttraumatic knee defects: long-term follow-up. Clin Orthop Relat Res. 2008;466(8):1863-70. 18465182 10.1007/s11999-008-0282-8 PMC2584250

[17] Frank RM Cotter EJ Strauss EJ Gomoll AH. Management of cartilage defects in the knee. J Bone Jt Surg Am. 2018;100:1181-95.

